# P21 Development of deep learning models for Sub-Cellular Fluctuation Imaging (SCFI): a 30 min antibiotic susceptibility test for urinary tract infections

**DOI:** 10.1093/jacamr/dlad077.025

**Published:** 2023-08-02

**Authors:** S Rama, M Antognozzi, W Szeremeta, K Phonrat, A Eley, C Bermingham, M Kyriakides, H Newman, K Bonney-Bhandal, J Preece, N Dorh, J Dorh

**Affiliations:** FluoretiQ Limited, Unit DX, St Philips Central, Albert Road, Bristol BS2 0XJ, UK; School of Physics, University of Bristol, Tyndall Avenue, Bristol, BS8 1TL, UK; School of Physics, University of Bristol, Tyndall Avenue, Bristol, BS8 1TL, UK; School of Physics, University of Bristol, Tyndall Avenue, Bristol, BS8 1TL, UK; School of Physics, University of Bristol, Tyndall Avenue, Bristol, BS8 1TL, UK; School of Physics, University of Bristol, Tyndall Avenue, Bristol, BS8 1TL, UK; FluoretiQ Limited, Unit DX, St Philips Central, Albert Road, Bristol BS2 0XJ, UK; FluoretiQ Limited, Unit DX, St Philips Central, Albert Road, Bristol BS2 0XJ, UK; FluoretiQ Limited, Unit DX, St Philips Central, Albert Road, Bristol BS2 0XJ, UK; FluoretiQ Limited, Unit DX, St Philips Central, Albert Road, Bristol BS2 0XJ, UK; FluoretiQ Limited, Unit DX, St Philips Central, Albert Road, Bristol BS2 0XJ, UK; FluoretiQ Limited, Unit DX, St Philips Central, Albert Road, Bristol BS2 0XJ, UK

## Abstract

**Background:**

We have developed a phenotypic and label-free antibiotic susceptibility test (AST) termed Sub-Cellular Fluctuation Imaging (SCFI) to address rising rates of antimicrobial resistance.^1^ SCFI is an advanced machine-learning enabled microscope that monitors real-time fluctuations of bacterial cell membrane in response to antibiotics. By quantifying changes in magnitude and location of light scattering caused by subcellular movement, we can detect metabolic changes that occur when bacteria are challenged with antibiotics.^2–5^ Here, we show that improvements to SCFI’s deep-learning models can correctly classify metabolic cell states for *Escherichia coli* (exponential, stationary, dead) and determine cell states for UTI-related species (*E. coli, Klebsiella pneumoniae* and *Proteus mirabilis*) with front-line antibiotics (trimethoprim and nitrofurantoin).

**Methods:**

A total of 100 μL per sample is introduced to microfluidic flow chambers and immobilized using a species-specific antibody coating for 10 min. The bacterial suspension is removed, washed (to minimize non-bound cells) and incubated with 100 μL of MHB broth containing either a treated (with antibiotic) or untreated (without antibiotic) condition for 30 min. Images are captured at a laser intensity of 20 Hz for 20 s, for ≥50 individual bacterial cells per test. Convolutional neural networks (CNN) were developed to enable classifications of bacteriostatic antibiotics (trimethoprim and methicillin) on *E. coli, K. pneumoniae* and *P. mirabilis* species to determine their respective performance metrics (sensitivity, specificity, PPV and NPV).

**Results:**

CNN models were developed to successfully classify treated, untreated, resistant and susceptible bacterial conditions. All conditions were tested in triplicate (*n*=150 cells) and demonstrated high accuracy (sensitivity 88%–98%, specificity 88%–99%, PPV 88%–99% and NPV 88%–98%) when compared with known MICs.

**Conclusions:**

In these experiments we successfully demonstrated rapid (30 min) and accurate (≥90%) classification of bacterial resistance states by deep learning techniques. These data also continue to support existing literature that SCFI is an AST that is agnostic to the antibiotic class and bacterial species used. This system is undergoing product development and will be translated into a bespoke hardware system for clinical and antibiotic research applications.
